# Earthquake prediction model using support vector regressor and hybrid neural networks

**DOI:** 10.1371/journal.pone.0199004

**Published:** 2018-07-05

**Authors:** Khawaja M. Asim, Adnan Idris, Talat Iqbal, Francisco Martínez-Álvarez

**Affiliations:** 1 Centre for Earthquake Studies, National Centre for Physics, Islamabad, Pakistan; 2 Department of Computer Sciences and IT, The University of Poonch, Rawalakot, Pakistan; 3 Department of Computer Science, Pablo de Olavide University, Seville, Spain; Northeast Normal University, CHINA

## Abstract

Earthquake prediction has been a challenging research area, where a future occurrence of the devastating catastrophe is predicted. In this work, sixty seismic features are computed through employing seismological concepts, such as Gutenberg-Richter law, seismic rate changes, foreshock frequency, seismic energy release, total recurrence time. Further, Maximum Relevance and Minimum Redundancy (mRMR) criteria is applied to extract the relevant features. A Support Vector Regressor (SVR) and Hybrid Neural Network (HNN) based classification system is built to obtain the earthquake predictions. HNN is a step wise combination of three different Neural Networks, supported by Enhanced Particle Swarm Optimization (EPSO), to offer weight optimization at each layer. The newly computed seismic features in combination with SVR-HNN prediction system is applied on Hindukush, Chile and Southern California regions. The obtained numerical results show improved prediction performance for all the considered regions, compared to previous prediction studies.

## 1 Introduction

Earthquakes are one of the major catastrophe and their unpredictability causes even more destruction in terms of human life and financial losses. There has been a serious debate about the predictability of earthquakes with two concurrent point of views related to their prediction. One school of thought considers it impossible phenomenon to predict while other have spent their resources and efforts to achieve this task. It is an undeniable fact that the seismologist community has been unsuccessful in developing methods to predict earthquakes despite more than a century of efforts. Earthquake prediction remained an unachieved objective due to several reasons. One of the reasons is the lack of technology in accurately monitoring the stress changes, pressure and temperature variations deep beneath the crust through scientific instruments, which eventually results in unavailability of comprehensive data about seismic features. The second probable cause is the gap between seismologists and computer scientist for exploring the various venues of technology to hunt this challenging task. With the advent of modern computer science based intelligent algorithms, significant results have been achieved in different fields of research, such as weather forecasting [1], churn prediction [2] and disease diagnosis [3]. Therefore, by bridging gap between computer science and seismology, substantial outcomes may be achieved.

Contemporary studies show that scientists have conducted earthquake prediction research through diverse techniques. Retrospective studies of various earthquake precursory phenomena show anomalous trends corresponding to earthquakes, such as sub-soil radon gas emission [4], total electron content of ionosphere [5], vertical electric field, magnetic field of earth [6] and so forth. The bright aspect of recent achievements is the encouraging results for earthquake prediction achieved through Computational Intelligence (CI) and Artificial Neural Networks (ANN) in combination with seismic parameters [7–12], thus initiating new lines of research and ideas to explore for earthquake prediction. There is a slight difference between earthquake prediction and earthquake forecasting. It is a continuously evolving concept with various definitions stated in literature [13]. In the view of authors, earthquake forecasting relates to the concept in which a certain probability of future earthquake occurrence is given. While prediction means that earthquake prediction is made in the form of Yes or No without any associated probability factor, irrespective of confidence in prediction.

Earthquake prediction problem is initially considered as a time series prediction [14] Later, seismic parameters are mathematically calculated on the basis of well-known seismic laws and facts, corresponding to every target earthquake (*E*_*t*_). The calculation of seismic parameters corresponding to every *E*_*t*_ provides a feature vector related to *E*_*t*_. Thus, earthquake prediction is carried out on the basis of computed features in place of time series of earthquakes, thereby converting a time series prediction problem into a classification problem. The mathematically calculated seismic parameters are basically meant to represent the internal geological state of the ground before earthquake occurrence. This research work employs the known mathematical methods and computes all the seismic features in a bid to retain maximum information, which leads to sixty seismic features corresponding to every earthquake occurrence (*E*_*t*_). After acquiring maximum available seismic features, Maximum Relevancy and Minimum Redundancy (mRMR) based feature selection is applied to select most relevant feature having maximum information. Support Vector Regression (SVR) followed by Hybrid Neural Network (HNN) model and Enhanced Particle Swarm Optimization (EPSO) is applied to model relationship between feature vectors and their corresponding *E*_*t*_, thereby generating robust earthquake prediction model (SVR-HNN). The distinctive contributions of this research methodology is summarized as:

- Sixty seismic features, calculated on the principle of retaining maximum information.
- A unique application of SVR in combination with HNN, on mRMR selected seismic features.

Hindukush, Chile and Southern California are three of the most active seismic regions in the world, thus considered in this study for studying problem of earthquake prediction. Earthquake prediction studies based on Artificial Neural Networks (ANN) have been performed on the considered seismic regions [9, 11, 15]. The proposed prediction methodology is separately applied to perform earthquake predictions for the said regions and results are evaluated. The suggested earthquake prediction model showed improved results as compared to the other models proposed for these regions.

The rest of the manuscript is structured as the section 2 contains literature survey. Section 3 details about the calculation of dataset, where section 4 explains the SVR-HNN based prediction mode. Results are evaluated and discussed in section 5.

## 2 Related literature

Numerous researchers have performed earthquake studies from prediction and forecasting perspectives through different angles. During earthquake preparation process beneath the surface, different geophysical and seismological processes occur. These happenings below the surface are believed to cause changes in sub-soil emission, vertical electric field and ionosphere. These precursory changes are studied and mapped retrospectively with major earthquakes [4, 5]. Earthquake prediction is also studied through observing behavioral changes in animals [16]. The animal behavioral study is carried out using motion-triggered cameras at Yanachaga National Park, Peru. The decline in animal activity has been observed prior to Contamana earthquake of magnitude 7.0 in 2011. However, the focus of this research is to study earthquake prediction through computational intelligence and machine learning based methods.

Algorithm M8 which aims to forecast the earthquake of magnitude 8.0 and above tested successfully at the different regions of the globe along with increased efficiency of intermediate term earthquake forecasting. The study analyzes the earthquake catalog and gives the alarming area in circular region for next five years [17, 18]. There are several studies conducted based on this algorithm and its advanced stabilized version i.e. M8S algorithm to forecast the seismic events of magnitude 5.5 and above [19].

The Three Dimensional Pattern Informatics (PI) approach is also applied which aims at the forecast of earthquakes with natural and synthetic data sets [20]. Considering the regional seismic environment, the method efficiently characterizes the spatial and temporal seismic activity patterns with angular allusion occurred in the extent of associated space. This technique is the improved version of two dimensional PI approach [21], in the sense that it resolves the vertically distributed seismic anomalies in the presence of complex tectonic structures. Moreover, it gives forecast by systematically analyzing the anomalous behaviors in the seismicity at regional level.

In another research work, the earthquake prediction for the regions of Southern California and San Francisco bay area have also been studied. Eight seismic parameters are mathematically calculated through the temporal sequence of past seismicity. The seismic parameters are then used in combination with Recurrent Neural Networks (RNN), Back Propagation Neural Network (BPNN) and Radial Basis Functions (RBF), separately. RNN yielded better results as compare to the other two applied neural networks [9]. Later, the Probabilistic Neural Network (PNN) has been applied for the same regions in combination with same seismic parameters [8], where PNN is reported to have produced better results for earthquakes of magnitude less than 6.0 than RNN. A similar approach with same eight seismic parameters has also been used to perform earthquake prediction for Hindukush region [11]. Pattern Recognition Neural Network (PRNN) is reported to have outperformed other classifiers, such as RNN, random forest, and LPBoost ensemble of trees for Hindukush region.

Earthquake magnitude prediction for northern Red Sea area is carried out in [22]. The methodology is based on the features extraction from the past available earthquake records followed by feedforward neural network. These features include sequence number of past earthquakes, respective locations, magnitude and depth. The similar kind of features have also been used for earthquake prediction for Pakistan region using BAT-ANN algorithm [23]. These features do not involve any seismological facts and laws, rather the direct modelling of earthquake sequence number, magnitude, depth and location with the future earthquakes is proposed.

Alexandridis et al. used RBF to estimate intervent time between large earthquakes of California earthquake catalog [24]. Aftershocks and foreshocks are removed from catalog through Reasenberg declustering technique before processing with neural network. Seismicity rates are taken as input to the neural network, whereas the intervent time between major earthquakes is taken as the output. Training of RBF is carried out through Fuzzy Mean algorithm.

The authors of [12, 15] proposed new mathematically computed seven seismic parameters to be used in combination with ANN to predict earthquakes in Chile and Iberian Peninsula. The methodology is capable of predicting seismic event of magnitude of 5.0 and above for horizon of 15 days. Further in [25], the results were improved by performing feature selection through Principle Component Analysis. Similarly, Zamani et al. [26] carried out retrospective studies for the September 10th, 2008 Qeshm earthquake in Southern Iran. The spatio-temporal analysis of eight seismic parameters is performed through RBF and Adaptive Neural Fuzzy Inference System (ANFIS). A sensitivity analysis of different geophysical and seismological parameters is performed in [27]. Results for earthquake prediction are obtained for the regions of Chile through varying combinations of parameters along with variations in training and testing samples.

Last et al. [10] performed earthquake prediction for Israel and its neighboring countries. The used earthquake data of past years has been treated to first clean foreshocks and aftershocks and then seismic parameters are calculated. The computed parameters are then employed for prediction in combination with Multi-Objective Info-Fuzzy Network (M-IFN) algorithm. The proposed prediction system is capable of predicting maximum earthquake magnitude and total number of seismic events for next year.

All the aforementioned methodologies study the earthquake prediction, while focusing only one region. The prediction models are not applied and tested to other earthquake prone regions and no comparisons are also carried out with the results of other research studies. In this research, the prediction model is applied to more than one region and comparisons are also drawn with the results, available in literature.

## 3 Regions selection and feature calculation

### 3.1 Region selection and earthquake catalog

In this study, three different regions, namely Hindukush, Chile, Southern California have been selected for prediction of earthquakes of magnitude 5.0 and above. The same regions selected in the precedent studies are also considered for this research [9, 11, 15]. The advantage of selecting the same regions is that results can be compared in the end, so as to prove the superiority of suggested methodology.

**Fig 1:**
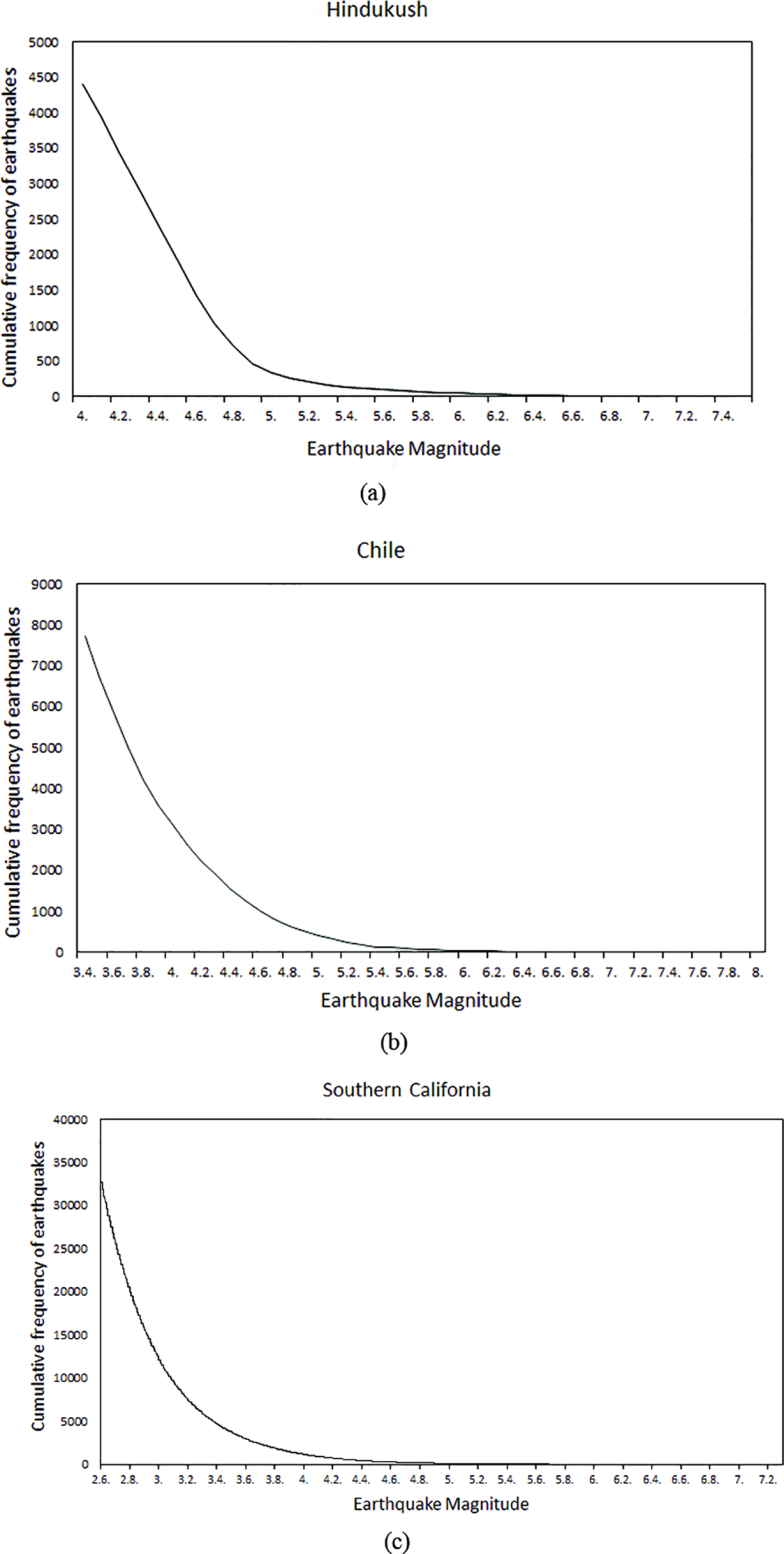
The curves demonstrates Gutenberg-Richter law, i.e. exponential rise in frequency of eathquakes with decreasing magnitude. (a): For Hindukush region catalog is complete upto M = 4.0 (b): Catalog of Chile shows completeness upto M = 3.4 (c): Southern California catalog is complete upto M = 2.6.

Earthquake catalogs of these regions have been obtained from United States Geological Survey (USGS) [28] for the period from January 1980 to December 2016. These catalogs are initially evaluated for cut-off magnitude. Cut-off magnitude corresponds to the earthquake magnitude in the catalog above which catalog is complete and no seismic event is missing. This depends upon the level of instrumentation. Dense instrumentation in a region leads to better completeness of catalog with low cut-off magnitude. The cut-off magnitude for Southern California region is found to be less than 2.6, for Chile it is 3.4 and for Hindukush it is 4.0. The completeness of magnitude for all three regions shows the density of instrumentation in these regions. There are different methodologies proposed in literature for evaluation of cut-off magnitude [29]. In this study, cut-off magnitude is determined through Gutenberg-Richter law. The point where curve deviates from exponential behavior is selected as a cut-off magnitude. All the events reported below cut-off magnitude are removed from the catalog before using for parameter calculation. Earthquake magnitudes and frequency of occurrences for each region is plotted as shown in Fig 1. The curves follow decreasing exponential behavior, which assures that each catalog is complete to its respective cut-off magnitude.

After parameter calculation a feature vector is obtained corresponding to every target earthquake (*Et*). In this study, earthquake prediction problem is designed/modeled as a binary classification problem. Every earthquake magnitude is converted to Yes, No (1, 0) through applying threshold on magnitude 5.0. It is too early in this field of research to predict actual magnitudes of future earthquakes; however, endeavors are on the way to predict the categories of future events.

**Fig 2:**
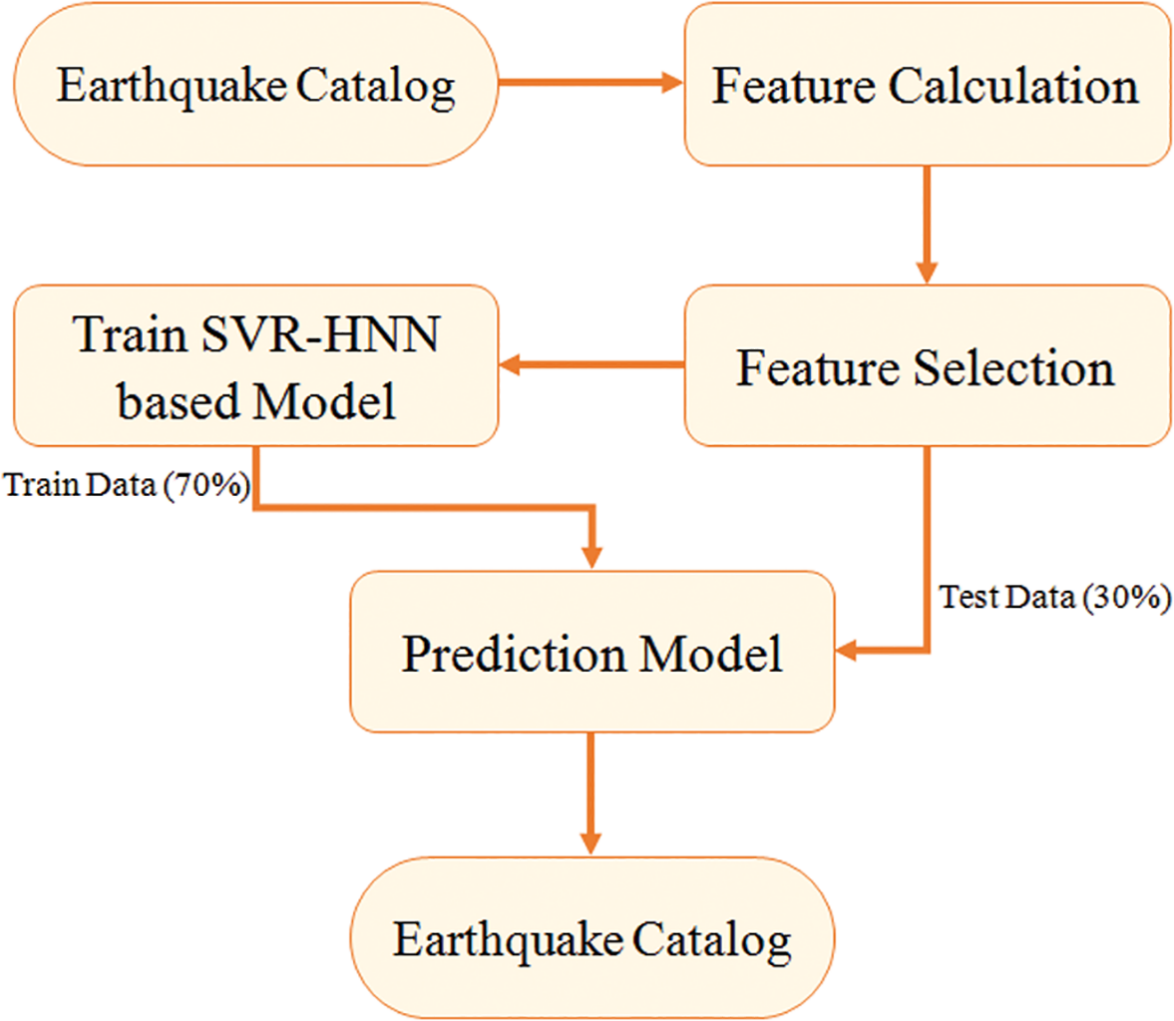
Flow chart of research methodology.

Fig 2 shows the overall flow of research methodology. The earthquake catalog is the starting point of this process therefore, quality of catalog directly affects the prediction results. Further processes involved are feature calculation, selection, training of model, and finally predictions are obtained on unseen part of dataset. In the end performance of prediction model is evaluated and comparison is drawn.

### 3.2 Parameter calculation

Features are the most important part of a classification problem. In this study, features are also referred as seismic parameters. These seismic parameters are calculated mathematically and are based upon well-known geophysical and seismological facts. There are different geophysical and seismic parameters suggested in literature for earthquake prediction studies employing computational intelligence [9, 15, 26]. Discovering new geophysical and seismological facts leading to earthquake prediction is another aspect of earthquake prediction studies, which is currently not included in the scope of this research work. Seismic parameters calculated in this research are broadly classified into two main categories with regards of calculation perspective, which are defined as:

- The seismic features whose calculation is not dependent upon any variable parameter are called non-parametric seismic features.
- The seismic features whose calculation is dependent upon any variable parameter, such as a threshold, are called parametric seismic features.

The important contribution of this research from the perspective of seismic parameters are:

1. All the available geophysical and seismological features employed for earthquake prediction in contemporary literature are taken into account simultaneously, which has never been done before.
2. Multiple values of parametric seismic features have been calculated based upon different variations of a variable parameter, in order to retain maximum available information about the internal geological state of the ground.

All the seismic features are calculated using the 50 seismic events before the event of interest (*E*_*t*_), which is to be predicted using the feature vector. The numbers of features reach to 60 in every instance according to suggested methodology. Later, in order to handle the issues related to curse of dimensionality, the feature selection technique based on Maximum Relevance and Minimum Redundancy (mRMR) is employed to choose the features having maximum relevant and discriminating information.

#### 3.2.1 Non-parametric seismic features

As the calculation of non-parametric seismic features is not dependent on any variable parameter thus such variables have one possible value for every instance.

- *a* and *b* value

These values are directly based on well-known geophysical law known as Gutenberg-Richter law. According to this law, number of earthquakes increase exponentially with decreasing magnitude, mathematically shown in Eq 1, where *N*_*i*_ is the total number of seismic events corresponding to Magnitude *M*_*i*_, *b* is slope of curve and a is y-intercept.

$$\log N_{i}=a-bM_{i}$$ (1)

The values of *a* and *b* are calculated numerically through two different methods. Eqs 2 and 3 represent the linear least square regression (*lsq*), while Eqs 4 and 5 show the maximum likelihood (*mlk*) method for calculation of *a* and *b* values. In earthquake prediction study for Southern California, linear least square regression analysis based method is proposed [9]. While maximum likelihood method is preferred for earthquake prediction for Chile [15].

$$b_{lsq}=\frac{\left(n\sum (M_{i}\log N_{i})-\sum M_{i}\sum \log N_{i}\right)}{\left((\sum M_{i}{)}^{2}-n\sum M_{i}{}^{2}\right)}$$ (2)

$$a_{lsq}=\sum \left(\log_{10}N_{i}+b_{lsq}M_{i}\right)/n$$ (3)

$$b_{mlk}=\frac{\log_{10}e}{mean(M)\text{-}\min(M)}$$ (4)

$$a_{mlk}=\log_{10}N+b_{mlk}\min(M)$$ (5)

- Seismic energy release

Seismic energy (*dE*) keeps releasing from ground in the form of small earthquakes as shown in Eq 6. If the energy release stops, the phenomenon is known as quiescence, which may release in the form of major event. State of quiescence may also lead to the reduction in seismic rate for the region, thereby decreasing *b* value.

$$dE^{\frac{1}{2}}=\frac{\sum {\left(10^{\left(11.8+1.5M\right)}\right)}^{\frac{1}{2}}}{T}$$ (6)

- Time of *n* events

Time (*T*) in days, during which *n* number seismic events have occurred before *E*_*t*_ as shown in Eq 7. In this study *n* is selected to be 50.

$$T=t_{n}-t_{1}$$ (7)

- Mean Magnitude

Mean magnitude (*M*_*mean*_) refers to the mean value of *n* events as shown in Eq 8. Usually the magnitude of seismic events rise before any larger earthquake.

$$M_{mean}=\frac{\sum_{i}M}{n}$$ (8)

- Seismic rate changes

Seismic rate change is the overall increase or decrease in the seismic behavior of the region for two difference intervals of time. There are two ways proposed to calculate seismic rate changes. z value shown in Eq 9 measures seismic rate change as proposed by [30], where *R1* and *R2* correspond the seismic rate for two different intervals. *S*_*1*_ and *S*_*2*_ represent the standard deviation of rate. *n*_*1*_ and *n*_*2*_ show the number of seismic event in both intervals.

$$z=\frac{R_{1}-R_{2}}{\sqrt{\frac{S_{1}}{n_{1}}+\frac{S_{2}}{n_{2}}}}$$ (9)

The other way for seismic rate change calculation is suggested in [31] and given in Eq.10, where, *n* represents total events in the whole earthquake dataset, *t* is total time duration and *δ* is the normalized duration of interest. *M(t*, *δ)* shows the number of events observed, defined using end time *t* and interval of interest *δ*. Both *z* and *β* values possess opposite signs and are independent from each other.

$$\beta =\frac{M(t,\delta )-n\delta }{\sqrt{n\delta (1-\delta )}}$$ (10)

- Maximum magnitude in last seven days

The maximum magnitude recorded in the days previous to *E*_*t*_ is also considered as an important seismic parameter as reported in [12, 15] and represented as x_6i_. The representation of this parameter is also kept the same as that of literature, so as to maintain better continuity. It is mathematically represented as given in Eq 11.

$$x_{6i}=\max\{M_{i}\},\;when\;t\in [\text{-}7,0)$$ (11)

Thus the total number of seismic parameters obtained from non-parametric features is accounted to 10.

#### 3.2.2 Parametric features

The formulae for parametric features contain a varying parameter, such as earthquake magnitude or *b* value. All these features are calculated through multiple available values of varying parameter. The details of all the parametric features are given below.

- Probability of earthquake occurrence

The probability of earthquake occurrence of magnitude greater than or equal to 6.0 is also taken as an important seismic feature. It is represented by x_7i_ and calculated through Eq 12. The inclusion of this feature supports the inclusion of Gutenberg-Richter law in an indirect way. The value of x_7i_ is dependent upon *b* value. Therefore, *b*_*lsq*_ and *b*_*mlk*_ are separately used to calculate x_7i_, thus giving two different values for this seismic feature.

$$x_{7i}=e^{\frac{-3b_{i}}{\log e}}$$ (12)

- Deviation from Gutenberg-Richer law

It is the deviation *η* of actual data from the Gutenberg-Richter inverse law as shown in Eq 13. This feature indicates how much actual data follows the inverse law of distribution. Its calculation is dependent upon *a* and *b* values, which in turn gives two values for *η*.

$$\eta =\frac{\sum {\left(\mathrm{logN}\text{-}a\text{-}\mathrm{bM}\right)}^{2}}{n\text{-}1}$$ (13)

- Standard deviation of *b* value

Standard deviation of *b* value *σb* is calculated using Eq 14. This feature is parametric because it is based upon the *b* value, which have two values, therefore adding two values of *σb*.

$$\sigma b=2.3b^{2}\sqrt{\frac{\sum_{i=1}^{n}{\left(M_{i}-\mathrm{mean}(M)\right)}^{2}}{n(n\text{-}1)}}$$ (14)

- Magnitude deficit

Magnitude deficit (*M*_*def*_) is the difference between the maximum observed earthquake magnitude and maximum expected earthquake magnitude (Eq 16). Maximum expected magnitude is calculated through Gutenberg-Richter’s law as given in Eq 15. The two sets of *a* and *b* values are separately used to calculate *M*_*def*._

$$M_{\max,\exp ected}=a/b$$ (15)

$$M_{def}=M_{\max,actual}-M_{\max,\exp ected}$$ (16)

- Total recurrence time

It is also known as probabilistic recurrence time (*T*_*r*_). It is defined as the time between two earthquakes of magnitude greater than or equal to *M*′ and calculated using Eq 17. This parameter is another interpretation of Gutenberg-Richter’s law. As evident from the statement of inverse law, there will be different value of *T*_*r*_ for every different value of *M*′, which would increase with increasing magnitude. Available literature does not focus on which value of *M*′ to be selected in such a scenario therefore *T*_*r*_ is calculated for every *M*′ from 4.0 to 6.0 magnitudes following the principle of retaining maximum available information. So for two sets of *a* and *b* values along with varying *M*′ adds 42 seismic features to the dataset.

$$T_{r}=\frac{T}{10^{a-bM^{\prime }}}$$ (17)

## 4 Earthquake prediction model

Unlike previous other simple earthquake prediction models proposed in literature, in this paper a multistep prediction model is suggested (SVR-HNN). It is a combination of various machine learning techniques with every technique complementing the other through knowledge acquired during learning. Thus, every step in this model is adding further improvements to the robustness therefore, resulting in a final improved version of prediction model. The layout of overall prediction model is given in Fig 2. Dataset obtained for all the three regions, is divided into training and testing sets. For training and validation purposes, 70% of the dataset is selected, while testing is performed on rest of 30% hold out dataset. The final results shown in Section 5 are the prediction results obtained on test dataset for every region, separately. The configurations and setups arranged in order to train a model are kept same for all three regions’ datasets. However, separate training has been performed for each region. The reason for separate training is that every region has different properties and can be classified tectonically into different categories, such as thrusting tectonics, strike-slip tectonics and so forth. Therefore every type of region possess different behaviors and relations to the earthquakes. Thus separate training for every region is meant to learn and model the relationship between seismic features and earthquakes for that particular region.

**Fig 3:**
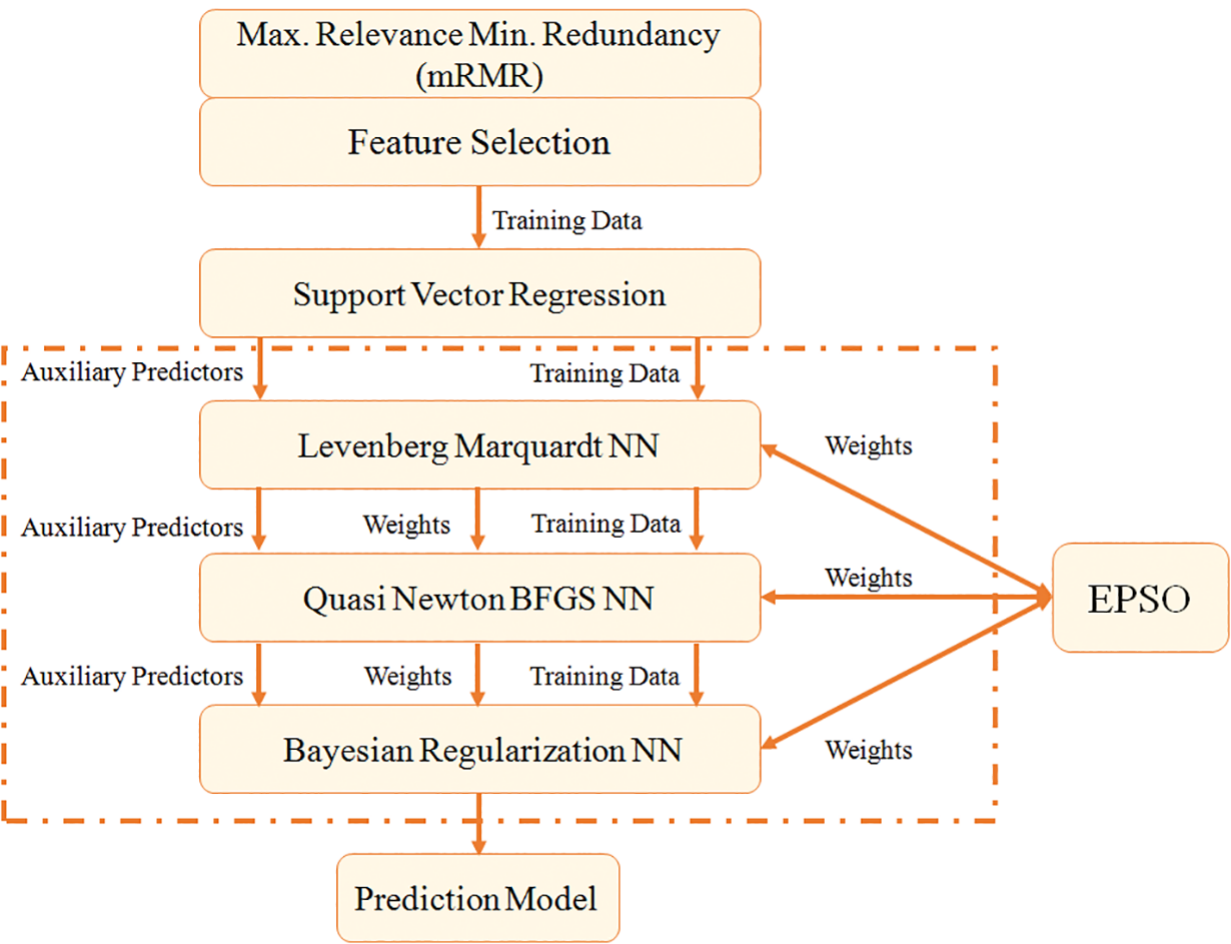
Flow chart of earthquake prediction model.

The proposed methodology includes the use of two step feature selection process. The features are selected after performing relevancy and redundancy checks, to make sure that only useful features are employed for earthquake prediction. The selected set of features are then passed to Support Vector Regression (SVR). The trend predicted by SVR is further used in combination with seismic features as input to the next stage of prediction model, i.e. ANN. The inclusion of SVR-output as a feature to ANN is to pass on the information learnt through SVR. After SVR, three different layers of neural networks are applied to the dataset in combination with Enhanced Particle Swarm Optimization (EPSO). The output of every ANN is used as an input to the next ANN in place of SVR-output along with feature set. The weight adjustments of each ANN layer is also passed to the next ANN, so that next ANN does not start learning from scratch. The purpose of including EPSO is to optimize the weights of ANN, which have tendency to get trapped in local minima. If during training ANN is stuck in local minima, EPSO plays a vital role in that scenario. The similar type of approach has also been used in other fields, such as wind power prediction [32, 33] with successful outcomes. The flowchart of earthquake prediction methodology is provided in Fig 3.

### 4.1 Feature selection

The total number of 60 seismic features are computed for every instance. The approach of feature calculation is useful that tends to gather maximum obtainable information however a reduced feature set can be selected for learning process instead of utilizing the complete set of 60 features. The features having less discriminating information or redundant information can be excluded. In order to deal with such a situation, two step feature selection is applied based on maximum relevance and minimum redundancy. After the calculation of all available features mRMR method selects the features having most relevant and discriminating information. This step also helps in avoiding the curse of dimensionality problem, which is a major issue in machine learning algorithms. Feature selection step is embedded as a part of prediction model because it is separately applied for each dataset. The different seismic regions considered in this study, represent varying seismic properties recorded as per calculated features and thus results in different seismic datasets. A feature in Hindukush dataset having insignificant information content may possess opposite trend for Chile or Southern California, which can actually be observed when feature selection is applied for all these regions. Therefore, it is inappropriate to declare features selected for a specific region to be best for all the other regions.

#### 4.1.1 Maximum relevance

Irrelevancy filter is first of the two steps of feature selection. It removes all the features that are irrelevant and having less information content to be useful for prediction. It is proposed in [34], while certain modifications in its formulation is suggested in [35]. This technique has already been used for feature selection in different classification problems, such as medical image processing, cancer classification. However, it has been used for the first time, for seismic feature selection. The methodology includes calculation of mutual information (MI) of every feature corresponding to target earthquakes (*E*_*t*_) in binary form. A suitable threshold is applied on MI and all the features having MI less than the threshold are ignored. The value of threshold is kept fixed in this model for the considered earthquake datasets.

Irrelevancy criterion filters out different features for all the three regions. For Hindukush region, 5 features are filtered out after this step and rest of 55 features are passed to the next step. Similarly for dataset of Chile, 4 features are excluded through irrelevance filter and 56 are passed to next step. In Southern California dataset, 55 features are considered fit for the next step after leaving out 05 features.

#### 4.1.2 Minimum redundancy

There are features which are showing redundant information in the dataset, therefore the inclusion of such features for earthquake prediction is of no use. Removing the features with redundant information is the second phase in feature selection. The idea of feature redundancy filter is proposed in [34] and certain changes in the implementation are given in [32]. The basic idea behind this technique is that MI is calculated in-between all the features. Any two features possessing maximum MI are considered to be having redundant information. Therefore a certain redundancy criterion (RC) is empirically selected and kept fixed in the learning process for all the three regions.

Like irrelevancy filter, this technique may show different results for every region. In Hindukush dataset, out of 55 relevant features, 32 are found to be redundant and left out, therefore, leaving behind 23 effective features. Similarly, Chile’s dataset contains 42 redundant features out of 56 relevant features and leaving behind 14 useful features and for Southern California dataset 25 useful features are selected after excluding 30 redundant features.

### 4.2 Support vector regression

SVR is a machine learning technique that learns in a supervised manner. It is first proposed in [36] and implementation is carried out using LIBSVM (A library for Support vector machine) [37]. SVR has wide range of applications for both classification as well as regression problems.

The model generated through training of SVR, gives predictions about an estimated earthquake magnitude corresponding to the feature vectors. SVR then imparts its knowledge of predicted earthquake magnitudes to HNN in next step through auxiliary predictions, to be used as a part of feature set. Experiments have proven that the auxiliary predictions from SVR when used in combination with features, adds a distinctive classification capability to the prediction model.

### 4.3 Enhanced Particle Swarm Optimization

There are different nature-inspired optimization algorithms in literature [38–44] however, EPSO has been employed in this study for weight optimization of ANN. EPSO is an evolutionary algorithm. The idea of particle swarm optimization (PSO) is given in [45]. In this optimization methodology, exploration is carried out for finding the best possible solution or position in the search space, like a bird or an organism searches for food. Different factors affect the hunt for best possible solution, like current position and velocity. The record of best local positon, global position and worst global position is also kept for generating optimized solution. EPSO is also a well-considered an optimization methodology, which is being used in different application fields and explained in detail in [32].

### 4.4 Hybrid Neural Networks

The next step of prediction model is to train hybrid neural network model. This step is the combination of three different ANNs along with optimization support extended from EPSO. The training is carried out through 1^st^ ANN and weights are then passed to EPSO for further optimization. In case, if ANN is stuck in local minima, EPSO has the capability to guide it out from this situation. The optimization measure for EPSO is set to Matthews Correlation Coefficient (MCC). If the ANN has already learnt the best possible relation between features and earthquakes, EPSO would return the same weight matrix. Thus, it can be said that EPSO is included in this methodology, to save ANNs from being trapped in local minima.

The training of this step is carried out for a binary classification problem, in which intention is to predict the earthquakes of magnitude 5.0 and above. Initially the dataset along with auxiliary prediction from SVR is passed to Levenberg-Marquardt neural network. The network trains in back propagation manner, where error at the output layer is back propagated in every epoch. When the training stops, the weight matrices are passed to EPSO along with training features and targets. EPSO optimizes the weights in terms of MCC and returns the optimized weights back to ANN. The predictions from optimized weights of EPSO are taken as auxiliary predictions in place of SVR-output to be used along with features for next ANN.

BFGS quasi-Newton backpropagation neural network (BFGSNN) is initialized with the weights of previously learnt NN. In this way, the already learnt information is transferred to the next NN along with auxiliary predictors. The network is trained similarly and the weights along with training data are passed to EPSO for optimization via MCC. The optimized weights are then used to initialize Bayesian Regularization Neural Network (BRNN) and training data is passed in combination with BFGSNN auxiliary predictors. EPSO is again employed to optimize the weights of NN. The pseudo-code is also included here for the consideration of the reviewer:

*Step by step procedure of SVR-HNN Model*:

**Inputs**: T, N, RF, SVR, HNN, EPSO

[**T** = Training Dataset

**N** = Numbers of layers of Neural Network

**RF** = Relevant Features

**SVR** = Support Vector Regresser

**NN** = Neural Network

**EPSO** = Enhanced Particle Swarm Optimization

**HNN** = Hybrid Neural Network (Combined package of NN+EPSO)]

    **RF** = mRMR (T)

    [SVR_Model, SVR_Predictions] = SVR[RF]

    **for** j = 1 to 3 (‘j’ indicates neural network and EPSO layer)

        **if** (j = = 1)(First NN takes SVR_Predictions as auxiliary input)

            [NN_j__Model, NN_j__Predictions] = NN_j_ [RF, SVR_Predictions]

                (EPSO optimizes NN, if it traps in local minima)

          [NN_j__Model, NN_j__Predictions] = EPSO_j_[RF, NN_j__Model, SVR_Predictions]

        **else**

            (Predictions of previous NN, becomes input to next NN)

          [NN_j__Model, NN_j__Predictions] = NN_j_ [RF, NN_j-1__Predictions]

          [NN_j__Model, NN_j__Predictions] = EPSO_j_[RF, NN_j__Model, NN_j-1__Predictions]

        **endif**

    **Endfor**

    SVR_HNN_Model = NN_3__Model (The model obtained after SVR and all layers of NN supported by EPSO)

    Performance Evaluation = SVR_HNN_Model[Test Dataset, Actual Labels]

In this work, the output of SVR is treated as its own opinion about the earthquake occurrence. Thus, in a bid to improve the performance of earthquake prediction a combination of three ANNs and EPSO, called as Hybrid Neural Network (HNN) is formulated. Seismic Features are passed to HNN for yielding the predictions. At this point of stage, the results obtained through SVR are considered as auxiliary predictions and passed on to HNN, along with other features. The opinion of SVR would also hold importance in terms of discriminating power between earthquake and non-earthquake occurrences. Therefore, when coupled with dataset of other seismic features, it highly improves the discriminating power of earthquake prediction, resulting in improved prediction performance of HNN. In HNN, the weights and outputs of each ANN is passed to the next layer of ANN, to make the consequent layer start learning ahead of a certain point. Thus, the feedback of SVR or ANN at a specific layer enhance the learning of HNN, leading to the improved earthquake prediction. The role of EPSO is to rescue ANN, when it is trapped in local minima through optimizing the weights. However, in case when ANN is performing well, the EPSO refrains from updating the weight. The model is trained using 70% of feature instances and later independent testing is performed on unseen 30% of data. In the training data two-third part is used for training the algorithm while one-third is used for validation of model. The evaluation of model is performed on unseen feature instances through considering well-known evaluation measures.

## 5 Results and discussion

**Fig 4:**
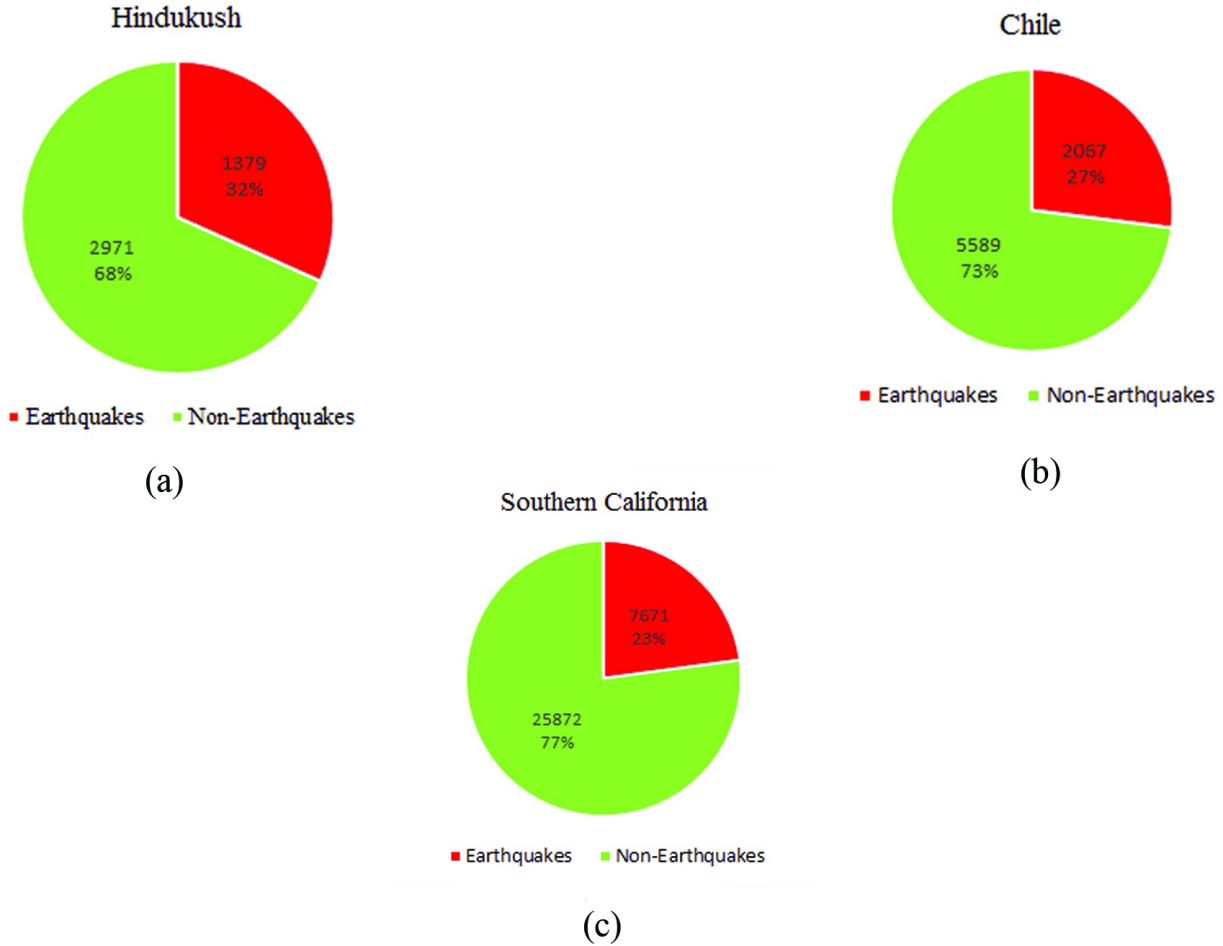
**Distribution of feature vectors corresponding to earthquakes and Non-Earthquakes in datasets for (a): Hindukush (b): Chile (c) Southern California**.

There are total 7656 feature instances for Chile out of which 2067 correspond to “Yes” earthquake while 5589 correspond to “No” earthquake. The total instances for Southern California are 33543 out of which 7671 belong to “Yes” earthquake while 25872 belong to “No” earthquake. Similarly, for Hindukush dataset, out of 4350 instances 1379 correspond to “Yes” while 2971 correspond to “No”. Therefore, the data distributions of the considered regions are highly imbalanced as shown in Fig 4.

### 5.1 Performance evaluation criteria

There are well-known performance metrics available for evaluation of binary classification results, such as sensitivity, specificity, positive predictive value or precision (P_1_), negative predictive value (P_2_), Matthews correlation coefficient (MCC) and R score. Whenever, predictions are made through an algorithm, it yields four categories of outputs, namely true positive (TP), false positive (FP), true negative (TP) and false negative (FN). TP and TN are the correct predictions made by the prediction model while FP and FN are the wrong predictions of the algorithm.

Sensitivity (S_n_) is the rate of true positive predictions out of all positive instances while specificity (S_p_) is the rate of true negative predictions out among all negative instances. P_1_ is the ratio of correct positive predictions out of all the positive predictions made by prediction model, whereas P_0_ refers to the correct negative predictions made by algorithm out of all negative predictions. P_1_ inversely relates to false alarms, which refers higher P_1_ means lesser false alarms and vice versa. Similarly, R score and MCC are also proposed as a balanced measure for binary classification evaluation. These are calculated using all four basic measures (TP, FP, TN, FN) and vary between -1 and +1. The values approaching +1 correspond to perfect classification, while 0 refers to total random behavior of prediction algorithm and -1 relates to opposite behavior of classification model. These two performance measures can be considered as a benchmark measure for drawing comparison, because the four types of basic measures are also incorporated in them as shown in Eqs 22 and 23. Accuracy is a very general criterion for evaluation, used in many perspectives. It states the overall accurate predictions made by the algorithm.

The purpose of analyzing the same results through these mentioned criteria is that each performance metric highlights certain aspect of results. Therefore, the purpose is to highlight all the merits and demerits of the results obtained through proposed prediction models. The formula for calculation of all the mentioned criteria are given in Eqs 18 to 24.

$$S_{n}=\frac{\mathrm{TP}}{\mathrm{TP}+\mathrm{FN}}$$ (18)

$$S_{p}=\frac{\mathrm{TN}}{\mathrm{TN+FP}}$$ (19)

$$P_{1}=\frac{\mathrm{TP}}{\mathrm{TP+FP}}$$ (20)

$$P_{0}=\frac{\mathrm{TN}}{\mathrm{TN+FN}}$$ (21)

$$\mathrm{MCC=}\frac{\mathrm{TPxTN-FPxFN}}{\sqrt{\mathrm{(TP+FP)(TP+FN)(TN+FP)(TN+FN)}}}$$ (22)

$$\mathrm{R=}\frac{\mathrm{(TPxTN)-(FPxFN)}}{\mathrm{(TP+FN)(FP+TN)}}$$ (23)

$$\mathrm{Accuracy=}\frac{\mathrm{TP+TN}}{\mathrm{TP+TN+FP+FN}}$$ (24)

### 5.2 Earthquake prediction results

SVR-HNN based model is separately trained for all three regions, using 70% of all the datasets. Prediction results are evaluated for all the regions. Accuracy is used for performance evaluation in many aspects. But in unbalanced classification problems, it may not be the best choice to only use accuracy for evaluation. When in a dataset, one class is in abundance and other is less, it is said to be unbalanced dataset. For example, in a dataset of 100 instances, if only 10 instances correspond to earthquake occurrence while rest of 90 belong to no-earthquake then a prediction algorithm with minimal prediction capability predicts all of them as no-earthquakes. In this scenario, the algorithm has no knowledge of predictability but its accuracy would still be 90%, therefore misguiding the overall capability of prediction model. However, MCC and R score would yield 0 value for this scenario, thereby giving better insights into the competence of prediction model. This is the reason different aspects of a prediction model is evaluated for better analysis. Earthquake prediction is highly delicate issue and false alarms may lead to financial loss and cause panic, therefore, cannot be tolerated. A prediction model with even less than 50% sensitivity but better P_1_ is preferable over another prediction model having around 90% sensitivity but lesser P_1._ Considering the fact that there exists no earthquake prediction system till date, thus results obtained through SVR-HNN model are commendable.

#### 5.2.1 Predictions for Hindukush region

Asim et al. [11] carried out earthquake prediction studies for Hindukush region where considerable prediction results are obtained through different machine learning techniques. Pattern recognition neural networks (PRNN) yields better results than other discussed methods, therefore PRNN is chosen in this work, for comparison with SVR-HNN based prediction methodology. Table 1 shows that SVR-HNN based prediction results are outperforming PRNN based predictions by wide margin in all aspects, except sensitivity. The value of MCC has been improved considerably from 0.33 to 0.6 and R Score from 0.27 to 0.58. Improvement is also observed in P_1_ from 61% to above 75%, thus improving false alarm generation for Hindukush region from 39% to less than 25%. Decreased sensitivity is acceptable with notable improvement in P_1_, MCC, R score and accuracy. A model with increased sensitivity may sensitize false earthquakes, leading to the generation of false alarms. Therefore, a model robust towards false alarms may have less sensitivity, which is acceptable given the other performance evaluation criteria have improved.

**Table 1:** Earthquake prediction results for Hindukush region.

Performance Evaluation	Asim et al. [11]	SVR-HNN
S_n_ (%)	91	69.6
S_p_ (%)	36	89.1
P_1_ (%)	61	75.4
P_0_ (%)	79	85.9
Acc (%)	65	82.7
MCC	0.33	0.60
R Score	0.27	0.58

#### 5.2.2 Predictions for Chile region

The results are even more improved for Chile region through SVR-HNN based prediction model. Previously, Reyes et al. [15] carried out earthquake prediction for four Chilean regions through applying different machine learning techniques with ANN achieving the best results. The ANN based results for all the Chilean regions are averaged (average of TPs, FPs, TNs, and FNs) in this study for drawing comparison with SVR-ANN based results. The proposed SVR-HNN approach has underperformed the ANN based Chilean results in the all aspects by considerable margins, except marginal difference in Specificity as evident from Table 2. False alarm generation reduced from 39% to less than 27% along with 5% increase in accuracy as well. The considerable difference can be observed in MCC and R score which increased from 0.39 to 0.61 and 0.34 to 0.60, respectively.

**Table 2:** Earthquake prediction results for Chile region.

Performance Evaluation	Reyes et al. [15]	SVR-HNN
S_n_ (%)	43.1	69.8
S_p_ (%)	91.3	90.5
P_1_ (%)	61.1	73.2
P_0_ (%)	83.5	89.0
Acc (%)	79.7	84.9
MCC	0.392	0.613
R Score	0.344	0.603

#### 5.2.3 Predictions for Southern California region

Southern California region is considered earlier for earthquake prediction using ANN by Panakkat and Adeli [9]. Recurrent Neural Network (RNN) is reported to have produced better results in terms of R Score. The results were evaluated in terms of false alarms ratio (FAR), Probability of detection (POD) or sensitivity, frequency bias (FB) and R score. To discuss the results through the same evaluation criteria, the values of basic performance parameters (TP, TN, FP and FN) are calculated through set of four equations of FAR, POD, FB and R Score. After calculating basic performance parameters, other evaluation criteria are calculated and given in Table 3. The results generated through SVR-HNN based methodology are better than RNN based results of [9]. False alarm generation are decreased considerably from 29% to less than 7%. A noteworthy increase in MCC and R score is also observed from 0.51 to 0.722 and .051 to 0.62, respectively. Hence proving the SVR-HNN based prediction methodology better than already available prediction models. SVR-HNN is outperforming previous prediction models because it is a multilayer model with every layer adding to its robustness. SVR provides initial estimation of the earthquake predictions, which is further refined by three different ANNs and EPSO supporting it through optimization.

**Table 3:** Earthquake prediction results for Southern California.

Performance Evaluation	Panakkat et al. [9]	SVR-HNN
S_n_ (%)	80	63.5
S_p_ (%)	71	98.7
P_1_ (%)	71	93.8
P_0_ (%)	86	90
Acc (%)	75.2	90.6
MCC	0.5108	0.722
R Score	0.5107	0.623

#### 5.2.4 Interregional comparison of earthquake prediction

**Fig 5:**
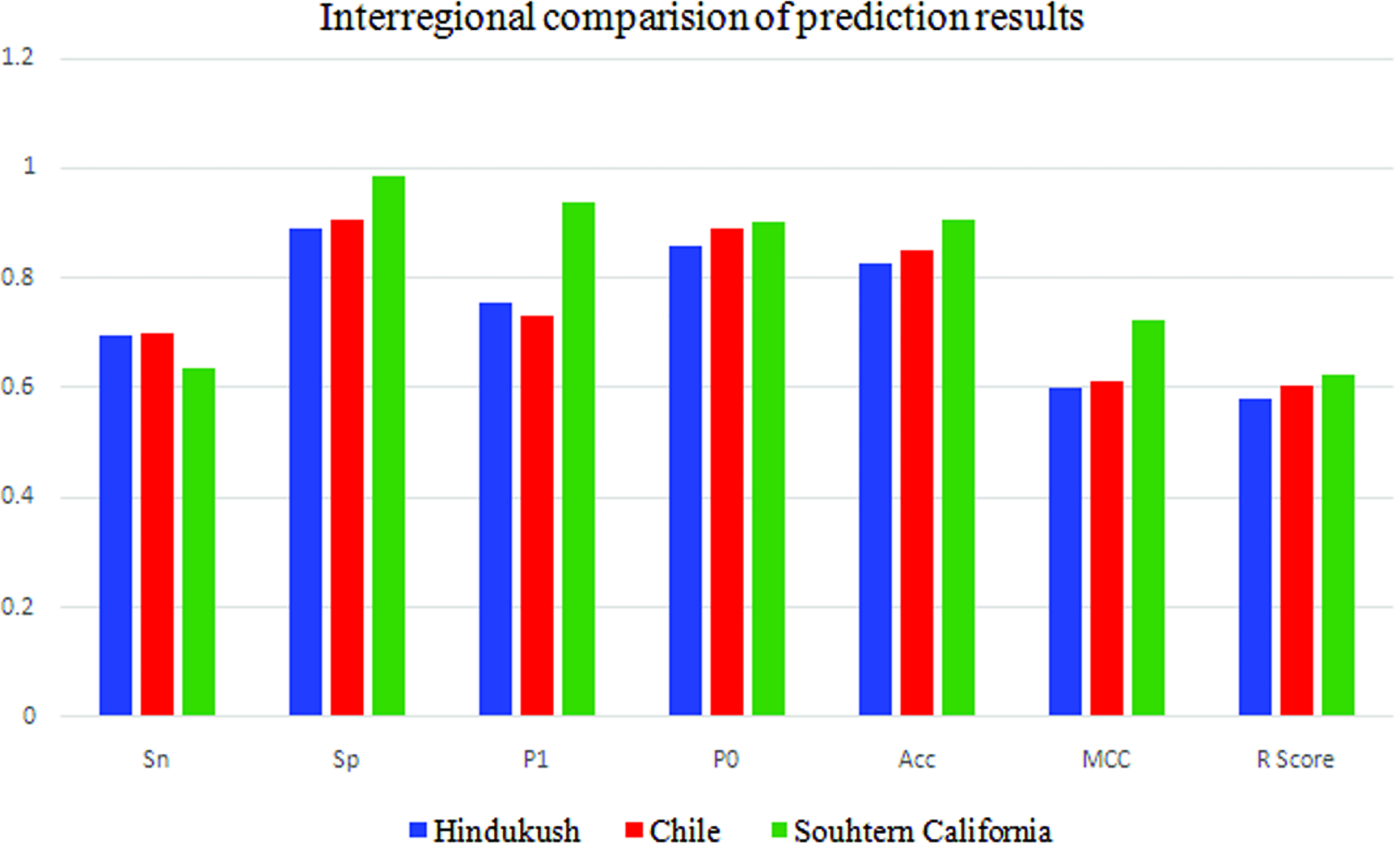
Interregional comparison of earthquake prediction results for three regions.

SVR-HNN based prediction model has shown improved results for Southern California as compared to the other two regions with 0.722, 0.623 and 90.6% of MCC, R score and accuracy, respectively. While Chilean region is holding second best position with MCC of 0.613, R score of 0.603 and accuracy of 84.9%. The SVR-HNN based proposed model has shown least results for the Hindukush region with MCC, R score and accuracy of 0.6, 0.58 and 82.7%, respectively. Fig 5 graphically compares the prediction results for all the three regions, scaled between 0 and 1. This inter-region results comparison has led to the practical visualization of the fact that lower cut-off magnitude and better completeness of earthquake catalog would lead to the improved results for earthquake prediction. The completeness magnitude of Southern California is taken as 2.6, for Chile its 3.4 and for Hindukush its 4.0. Hence demonstrated that earthquake prediction results are inversely related to the cut-off magnitude. In other words, it can be inferred that dense instrumentation for earthquake monitoring plays key role for better earthquake prediction.

The overall achievements of this study are presented as:

1. SVR-HNN based prediction methodology generated considerably improved results for Hindukush, Chile and Southern California. It has exceedingly outperformed other available predictions results in literature.
2. Inter-regional comparison of prediction results shows that the region with better maintained earthquake catalog having low cut-off magnitude is capable of generating better prediction results. It highlights the need of better instrumentation for earthquake catalog maintenance.

#### 5.2.5 Comparison between individual techniques and SVR-HNN

In order to prove the superiority of SVR-HNN model, the earthquake prediction performance is also computed separately using SVR and HNN. An independent arrangement has been made to get the earthquake prediction results for SVR and HNN. The performance of SVR and HNN is compared with the combine results of both (SVR-HNN). A notable difference between the performances of individual techniques and their combination can be observed. MCC of 0.43 and 0.41 is obtained for Hindukush region using SVR and HNN, respectively. The combination of two techniques by including SVR as auxiliary predictor for HNN improves MCC to 0.58. A similar trend in improvement is also observed for other performance measures, as shown in Table 4, for the three considered regions. Thereby, proving the superiority of SVR-HNN over their separate counter parts.

**Table 4:** Performance comparison of SVR, HNN with SVR-HNN.

Region	Hindukush			Chile			Southern California		
Criteria	SVR	HNN	SVR-HNN	SVR	HNN	SVR-HNN	SVR	HNN	SVR-HNN
S_n_ (%)	50.0	0.541	69.9	43.7	54.7	69.8	55.8	55.0	63.5
S_p_ (%)	89.0	85.4	89.1	93.6	92.4	90.5	98.1	97.2	98.7
P_1_ (%)	69.2	64.0	75.4	72.3	73.8	73.2	89.9	86.8	93.8
P_0_ (%)	78.3	79.4	85.9	81.4	83.9	89.0	87.9	87.9	90.0
Acc (%)	76.1	75.2	82.7	79.9	81.8	84.9	88.2	87.7	90.6
MCC	0.43	0.41	0.6	0.44	0.52	0.613	64.6	0.62	0.722
R Score	0.39	0.39	0.58	0.37	0.47	0.603	53.7	0.52	0.623

The role of EPSO is to optimize the weights of neural networks. Artificial neural networks have tendency of getting trapped in local minima. If such a situation happens during the training of a neural network, EPSO helps ANN in escaping local minima and guides in towards optimum solution. But this is not the case every time that ANN is trapped in local minima. It may happen occasionally. If ANN is already performing well and learning in the right direction, the inclusion of EPSO does not affect the weights of ANN in such a situation. The term HNN stands for combination of three different neural networks coupled with EPSO. Sometimes HNN without EPSO would show the similar results as compare to HNN with EPSO, however, on some other training occasions, it may show lesser performance.

#### 5.2.6 Performance stability

**Fig 6:**
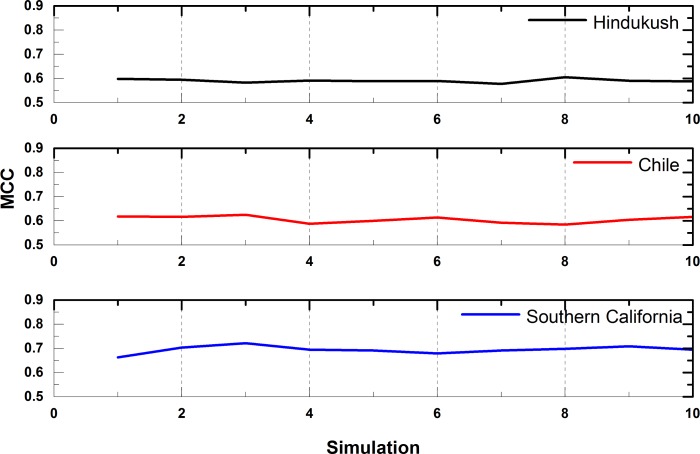
Performance of SVR-HNN over multiple runs of simulation.

The performance of SVR, HNN and EPSO are dependent on respective parameter selection. Thus, the collective performance obtained in SVR-HNN model is governed through selection of appropriate parameter values. The extensive experimentation is performed to empirically select the values for parameters, which obtain good results for each of the used algorithms (SVR, HNN, and EPSO). The proposed model has shown consistent performance for the three regions, which approves the appropriate selection of values for parameters. The ten simulation runs of SVR-HNN model show little variation, which strengthens the claim of appropriate selection of parametric values, leading to a stable earthquake prediction performance in three regions. The graphs given in Fig 6 show, the performance stability of SVR-HNN model for the three considered regions.

## Conclusions

In this study, interdisciplinary research has been carried out for earthquake prediction through interaction of seismology-based earthquake precursors and computer science based computational intelligent techniques. A robust multilayer prediction model is generated in combination with the computation of maximum obtainable seismic features. Sixty seismic features are computed for Hindukush, Chile and Southern California regions. Separate feature selection is performed for every region through maximum relevance and minimum redundancy (mRMR) approach. The selected features are employed for training of an earthquake prediction model. The prediction model consists of Support Vector Regressor (SVR) followed Hybrid Neural Networks (HNN) and Enhanced Particle Swarm Optimization (EPSO). SVR provides an initial estimation for earthquake prediction which is passed to HNN as an auxiliary predictor in combination with features. Three different neural networks are further employed along with EPSO weight optimization. Thus SVR-HNN based prediction model is trained and tested successfully with encouraging and improved results for all three regions.
